# Urocortin-1 Is Chondroprotective in Response to Acute Cartilage Injury via Modulation of Piezo1

**DOI:** 10.3390/ijms23095119

**Published:** 2022-05-04

**Authors:** Rebecca C. Jones, Kevin M. Lawrence, Scott M. Higgins, Stephen M. Richardson, Paul A. Townsend

**Affiliations:** 1 Manchester Cancer Research Centre, Division of Cancer Sciences, Faculty of Biology, School of Medical Sciences, Medicine and Health, University of Manchester, Wilmslow Road, Manchester M20 4GJ, UK; beckycatrin2@gmail.com (R.C.J.); drkevin60lawrence@gmail.com (K.M.L.); scott.higgins-2@postgrad.manchester.ac.uk (S.M.H.); 2 Division of Cell Matrix Biology and Regenerative Medicine, School of Biological Sciences, Faculty of Biology, Medicine and Health, Manchester Academic Health Science Centre, University of Manchester, Oxford Road, Manchester M13 9PT, UK; 3 School of Biosciences and Medicine, Faculty of Health and Medical Sciences, University of Surrey, Guildford GU2 7XH, UK

**Keywords:** post-traumatic osteoarthritis, osteoarthritis, chondrocyte, chondroprotection, cell survival, loading, injury, explant, urocortin

## Abstract

Post-traumatic OA (PTOA) is often triggered by injurious, high-impact loading events which result in rapid, excessive chondrocyte cell death and a phenotypic shift in residual cells toward a more catabolic state. As such, the identification of a disease-modifying OA drug (DMOAD) that can protect chondrocytes from death following impact injury, and thereby prevent cartilage degradation and progression to PTOA, would offer a novel intervention. We have previously shown that urocortin-1 (Ucn) is an essential endogenous pro-survival factor that protects chondrocytes from OA-associated pro-apoptotic stimuli. Here, using a drop tower PTOA-induction model, we demonstrate the extent of Ucn’s chondroprotective role in cartilage explants exposed to excessive impact load. Using pathway-specific agonists and antagonists, we show that Ucn acts to block load-induced intracellular calcium accumulation through blockade of the non-selective cation channel Piezo1 rather than TRPV4. This protective effect is mediated primarily through the Ucn receptor CRF-R1 rather than CRF-R2. Crucially, we demonstrate that the chondroprotective effect of Ucn is maintained whether it is applied pre-impact or post-impact, highlighting the potential of Ucn as a novel DMOAD for the prevention of injurious impact overload-induced PTOA.

## 1. Introduction

Injurious, high-impact loading events to weight-bearing joints, such as those occurring in the knee during accidents or sporting injuries, often cause cartilage damage, as well as high levels of chondrocyte cell death and a phenotypic shift in the remaining cells towards a more catabolic state [1,2,3,4]. The long-term consequence of this damage and associated changes in cell number and phenotype is post-traumatic osteoarthritis (PTOA), which accounts for approximately 12% of all OA cases [5]. PTOA disproportionately affects young active individuals, with 23–50% of those suffering a traumatic knee injury developing PTOA and diagnosis occurring 10 years earlier than those without any history of injury [6,7,8]. In the absence of disease-modifying OA drugs (DMOADs), aside from any analgesic intervention [9] or dietary adjustment, symptoms and pain will persist until joint replacement surgery is performed [5].

As chondrocytes play an important role in the homeostasis of articular cartilage, their survival is crucial for the maintenance of tissue integrity. However, within the avascular and aneural environment of the cartilage, the paucity of nutrients means that replication of chondrocytes is extremely limited, and hence their loss is permanent. The lack of macrophages within cartilage also means dead cells and their remnants remain within the tissue, inducing a further degradative cascade [10,11]. As such, it is hypothesised that any potential DMOAD with the ability to protect chondrocytes in situ, and thereby prevent further damage, would be highly desirable.

We have previously demonstrated that the short endogenous peptide urocortin-1 (Ucn) and its relevant G-protein coupled receptor (corticotrophin releasing factor receptor 1 (CRF-R1)) are expressed in chondrocytes, where Ucn can function as both an essential endogenous pro-survival factor and protect from OA-associated pro-apoptotic stimuli [12,13]. We have determined in vitro that the mechanism by which Ucn exerts its pro-survival effect on chondrocytes involves blockade of the mechanosensitive ion channel, Piezo1 [13]. Piezos represent a recently cloned novel class of non-selective cation channels which can detect and transduce mechanical stresses into an inward Ca^2+^ conductance [14,15]. If left unchecked, this ultimately results in Ca^2+^ overload and chondrocyte cell death [16,17]. Having identified the ion channel Piezo1 as the target for Ucn-mediated chondroprotection, we hypothesised that Ucn may protect chondrocytes from traumatic cartilage injury associated with PTOA.

Previously used models have replicated the force and impact experienced by chondrocytes within the joint. These have included 3D models to study OA [18,19], where hydrogels were seeded with primary chondrocytes [20]. These methodologies have several disadvantages, as the chondrocytes are removed from their native environment and, therefore, interactions between the cells and their pericellular or extracellular environment are lost. Additionally, the cell extraction process from cartilage is harsh and may result in altered cell signalling. Ex vivo models of OA do not encounter such difficulties and are particularly useful for the study of PTOA. Several studies have applied a single impact from a drop tower device, which represents an efficient means of modelling pathological impact [3,21,22,23]. This method has been successfully used to impact equine cartilage as a means of modelling PTOA and chondrocyte cell death arising from excessive calcium influx [23].

To extend our previous in vitro studies, we have developed an ex vivo model of PTOA, whereby porcine cartilage explants are subjected to various impact events administered from a drop tower. This has allowed us to investigate the extent of Ucn’s chondroprotective role in response to excessive impact load and, by use of Piezo1-modifying agents, enabled us to confirm a role for Piezo1 in this chondroprotective effect.

## 2. Results

### 2.1. Dose Response of Impact on Cell Death in Porcine Cartilage Explants

**Figure 1:**
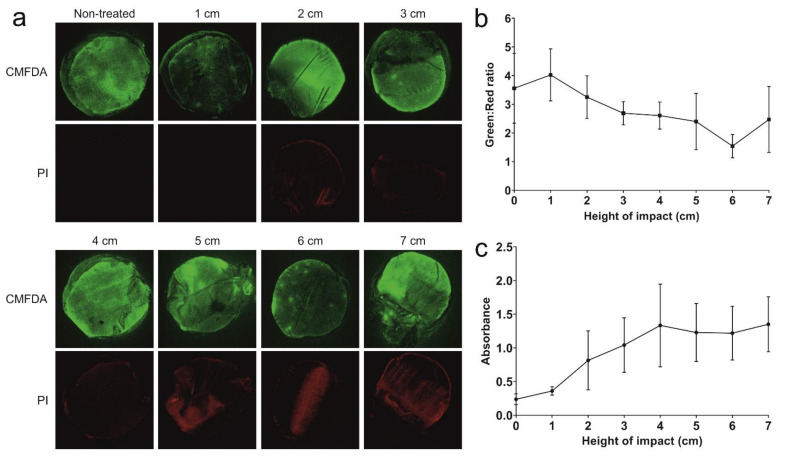
**Establishment of a single-impact trauma model for porcine explants.** (**a**) Representative macro-confocal images of porcine articular cartilage explants 72 h after impact with a 500 g weight dropped from varying heights. Viable cells shown in green (CMFDA) and dead cells in red (PI). (**b**) Quantification of green:red (live:dead) ratio from impact experiments shown in (**b**) (*n* = 3 explants). (**c**) LDH release from explants 72 h after impact with a 500 g weight dropped from varying heights (*n* = 3 explants). Graphs show mean +/− SEM.

To confidently use the custom drop tower for downstream applications, it was first necessary to optimise the energy of impact. Explants were impacted from increasing heights at 1 cm intervals from 1 cm to 7 cm and visualised with CMFDA (green) and PI (red) staining at 72 h after impact under a macro-confocal microscope (Figure 1a). Increasing heights of impact resulted in a decreasing trend of green:red ratio, although this was not significant between no impact and any height of impact (Figure 1b). LDH in the media at 72 h also increased with greater heights, although not significantly (Figure 1c). LDH release seemed to stabilise after 5 cm impact. Based on these observations, an impact height of 5 cm was selected, as it was sufficient to induce reproducible levels of cell death.

### 2.2. Pre-Impact Addition of Ucn Prevents Impact-Induced Cell Death

**Figure 2:**
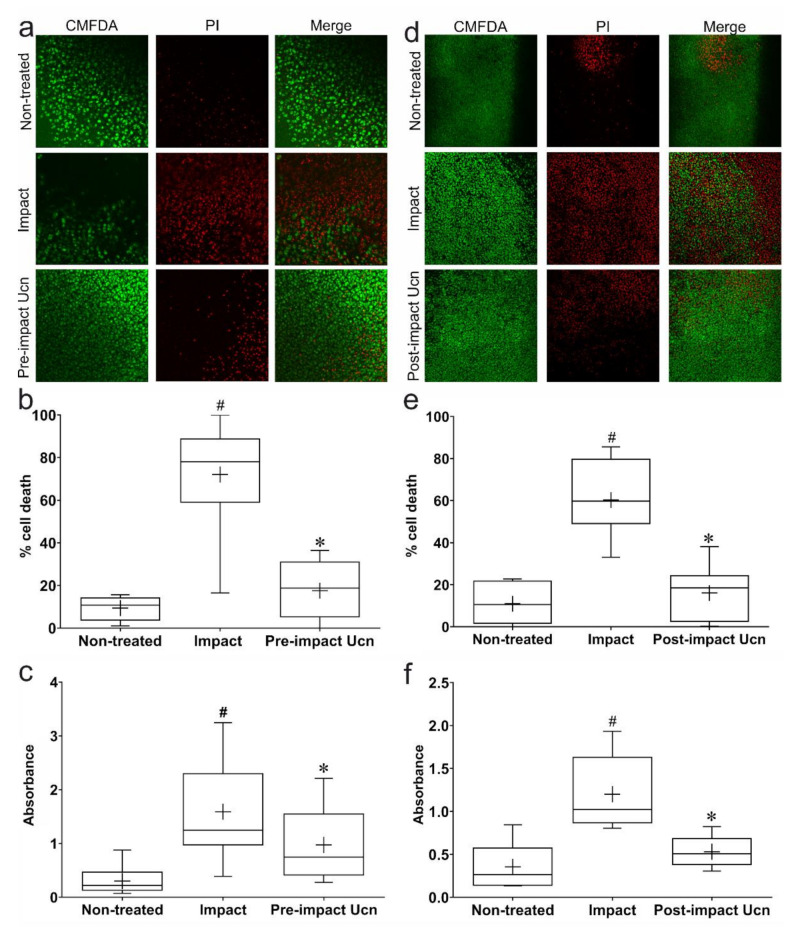
**Pre-treatment and post-treatment of porcine articular cartilage with Ucn reduces trauma-induced cell death.** Representative confocal images of porcine articular cartilage explants 72 h post-impact with or without Ucn pre-treatment (**a**) or post-treatment (**d**). Viable cells shown in green (CMFDA) and dead cells in red (PI). Quantification of percentage cell death 72 h post-impact with or without Ucn pre-treatment (**b**) or post-treatment (**e**) (*n* = 6 explants, 5 fields of view/explant; # = *p* < 0.01 vs. non-treated, * = *p* < 0.01 vs. impact). LDH absorbance readings from media collected at 72 h post-impact with or without Ucn pre-treatment (**c**) or post-treatment (**f**) (*n* = 6 explants; # = *p* < 0.01 vs. non-treated, * = *p* < 0.01 vs. impact).

Having defined an appropriate height of impact, the effect of Ucn on impacted explants was investigated. Ucn-treated explants were impacted 30 min after the addition of Ucn and cultured for an additional 72 h before visualisation on a confocal microscope (Figure 2a). Impacted explants without Ucn pre-treatment demonstrated a significant increase in the percentage of PI-stained cells when compared to non-treated explants (Figure 2b). This effect was significantly rescued by pre-impact treatment of explants with Ucn. An LDH assay conducted at 72 h also showed an increase in its release to the surrounding media for impacted explants without Ucn pre-treatment. This increase was prevented by Ucn pre-treatment, which showed a significant decrease in LDH release, bringing the absorbance down to levels comparable to controls (Figure 2c).

### 2.3. Post-Impact Addition of Ucn Prevents Impact-Induced Cell Death

To provide a more representative model of PTOA in terms of a potential therapy, it was also necessary to investigate the effect of Ucn administration post-impact. Explants were impacted and incubated for 30 min in normal 1% FCS media, before the addition of 0.1 µM Ucn to the media, and imaged at 72 h post-impact (Figure 2d). Impact again caused a significant increase in the percentage of PI-stained dead cells and LDH release compared to controls. Post-impact Ucn-treated explants visualised 72 h after trauma exhibited a significant decrease in the percentage of PI-stained dead cells when compared to impact alone (Figure 2e). This result was also reflected in levels of LDH release, where Ucn-treated explants showed significantly lower absorbance values than those seen in impacted explants alone. No significant difference in the percentage of dead cells or LDH release was observed between non-treated controls and impacted explants treated with Ucn post-impact (Figure 2f).

### 2.4. Chondroprotection in Response to Impact Is Mediated through CRF-R1

**Figure 3:**
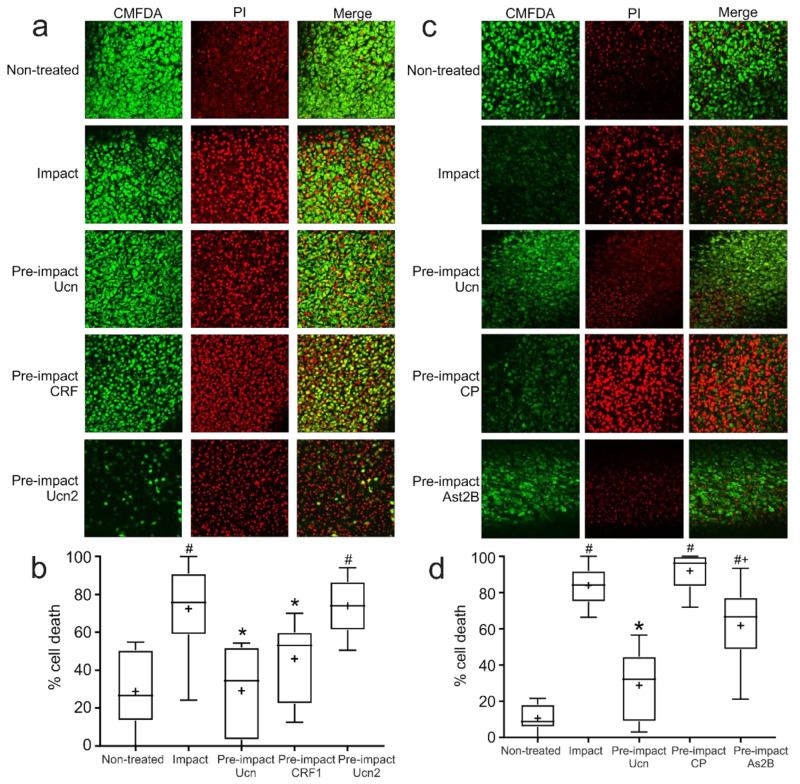
**Impact-induced chondrocyte cell death is Ucn receptor subtype-specific**. (**a**) Representative confocal microscopy images of explants 72 h post-impact, pre-treated with agonists of CRF-R1 (Ucn-1 and CRF) or CRF-R2 (Ucn-1 and Ucn-2). Live cells were stained with CMFDA (green) and dead cells with propidium iodide (red). (**b**) Quantification of percentage cell death from impact experiments shown in (**a**) (*n* = 6 explants, 5 fields of view/explant; # = *p* < 0.01 vs. non-treated, * = *p* < 0.01 vs. impact). (**c**) Representative confocal microscopy images of explants 72 h post-impact, pre-treated with antagonists of CRF-R1 (CP) or CRF-R2 (Ast2B). Live cells were stained with CMFDA (green) and dead cells with PI (red). (**d**) Quantification of percentage cell death from impact experiments shown in (**c**) (*n* = 6 explants, 5 fields of view/explant; # = *p* < 0.05 vs. non-treated, * = *p* < 0.01 vs. impact, + = *p* < 0.05 vs. pre-impact CP).

Because Ucn has a similar affinity to both CRF-R1 and CRF-R2, and as both receptors are expressed by chondrocytes, it was important to determine which receptor was responsible for its chondroprotective effect. CRF is a specific agonist for CRF-R1, whereas Ucn2 and Ucn3 both interact more selectively with CRF-R2 [24]. Therefore, here we compared the chondroprotective effects of pre-treatment with CRF or Ucn2 alongside Ucn to fully determine the receptor subtype involved (Figure 3a). Both Ucn and CRF significantly reduced levels of cell death induced by impact at 72 h. However, Ucn2 had no effect on cell viability, suggesting that an interaction with CRF-R1 is necessary for the observed chondroprotective effect (Figure 3b).

To further assess whether this chondroprotection was a direct ligand interaction with CRF-R1 specifically, selective antagonists to the receptors were employed. These were used to treat explants pre-impact to block any potential rescue effect of Ucn (Figure 3c). Explants treated with CP, a specific antagonist of CRF-R1, produced consistently higher levels of cell death than non-treated controls. Explants treated with the CRF-R2 antagonist Ast2B displayed significantly lower levels of cell death than CP; however, the cell death was still significantly higher than that seen in non-treated or Ucn-treated explants (Figure 3d). This suggests that there may be some protective role of Ucn mediated via binding to CRF-R2, although at a lower level than that seen in CRF-R1 binding.

### 2.5. Pre- and Post-Impact Addition of Ucn Reduces Intracellular Calcium Level

**Figure 4:**
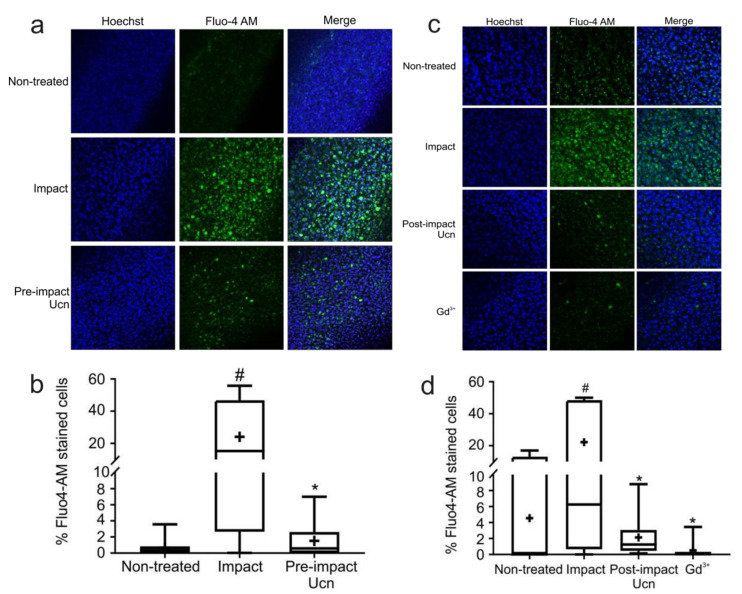
**Chondroprotection by Ucn involves the prevention of Ca^2+^ overload and blockade of a non-selective cation channel.** (**a**) Representative images of explants 1 h post-impact with or without Ucn pre-treatment. Intracellular calcium stained with Fluo-4 AM (green) and nuclei with Hoechst-33258 (blue). (**b**) Quantification of Fluo-4 AM-stained cells from experiments shown in (**a**) (*n* = 6 explants, 5 fields of view/explant; # = *p* < 0.05 vs. non-treated, * = *p* < 0.01 vs. impact). (**c**) Representative images of explants 1 h post-impact with or without Ucn or Gd^3+^ treatment. Intracellular calcium stained with Fluo-4 AM (green) and nuclei with Hoechst-33258 (blue). (**d**) Quantification of Fluo-4 AM-stained cells from experiments shown in (**c**) (*n* = 6 explants, 5 fields of view/explant; # = *p* < 0.05 vs. non-treated, * = *p* < 0.01 vs. impact).

Previous work on chondrocyte cell lines and primary human chondrocytes has identified a link between increased intracellular calcium levels post-impact and cell death [23,25,26,27]. To understand if this was the case in our system, the effect of Ucn was investigated using the previously described drop tower model. Explants were treated with Ucn 30 min prior to impact and visualised at 1 h post-impact (Figure 4a). Impacted explants had high levels of Fluo-4 AM-stained cells compared to non-treated explants. However, Ucn pre-treated explants exhibited significantly lower levels of Fluo-4 AM-stained cells (Figure 4b).

This experiment was repeated with a 30 min post-impact dose of Ucn or Gd^3+^ to compare the effect of a non-selective cation channel blocker c). Both Gd^3+^ and post-impact Ucn treatment were able to significantly reduce the number of Fluo-4 AM-stained cells at 1 h post-impact when compared to explants impacted alone (Figure 4d). This strongly implicated a non-selective cation channel in this Ucn-mediated chondroprotection following impact.

### 2.6. The TRPV4-Specific Blocker HC-067047 Does Not Prevent Impact-Induced Cell Death

**Figure 5:**
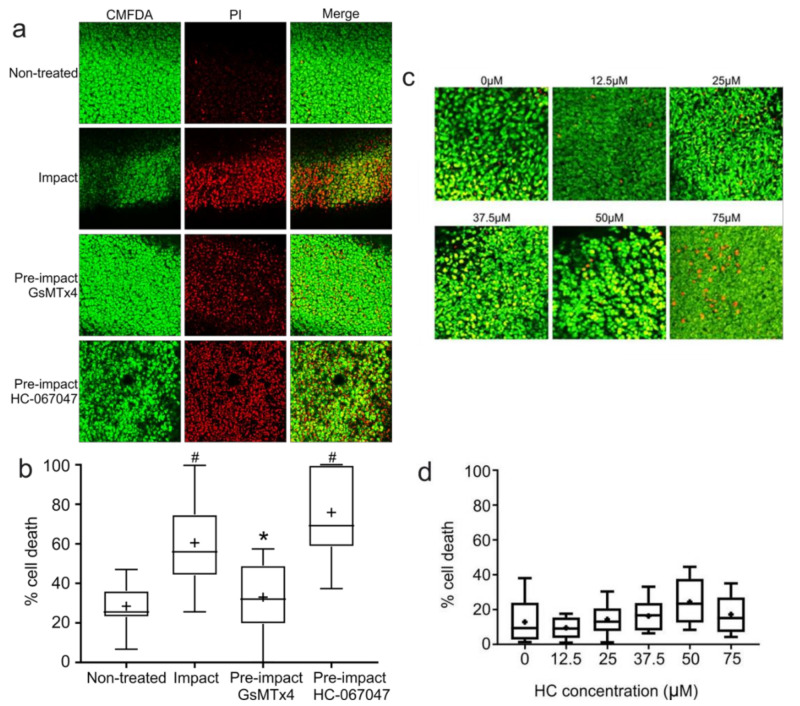
**TRPV4 is not associated with impact-induced cell death.** (**a**) Representative images of explants 72 h following impact treated with GsMTx4 or HC-067047 prior to impact. Live cells stained with CMFDA (green) and dead cells with PI (red). (**b**) Quantification of percentage cell death from impact experiments shown in (**a**) (*n* = 6 explants, 5 fields of view/explant; # = *p* < 0.01 vs. non-treated, * = *p* < 0.01 vs. impact). (**c**) Representative images of explants 72 h following treatment with increasing doses of HC-067047. Live cells stained with CMFDA (green) and dead cells with propidium iodide (red). (**d**) Quantification of percentage cell death from experiments shown in (**c**) (*n* = 3 explants, 3 fields of view/explant). There were no significant differences between levels of cell death in any condition.

Much of the literature surrounding the role of mechanosensitive ion channels on chondrocytes focuses on transient receptor potential vanilloid 4 (TRPV4) and Piezo1 and 2. In the absence of specific blockers for the Piezo channels, the chondroprotective effect of the spider venom GsMTx4 (a non-selective cationic mechanosensitive ion channel blocker effective against Piezo1 but not TRPV4) [28,29] was compared to that of the TRPV4-specific channel blocker HC-067047, to determine which of these channels was most likely to be linked to the chondroprotection observed following explant impact. Explants were pre-treated with either GsMTx4 or HC-067047 for 30 min prior to impact and cell viability was assessed at 72 h post-impact (Figure 5a). While explants pre-treated with GsMTx4 were able to significantly reduce levels of impact-induced cell death, there was no rescue effect observed in the explants pre-treated with HC-067047 (Figure 5b).

To confirm that the increase in cell death observed in HC-067047-treated explants was due to a lack of chondroprotection and not a cytotoxic effect of the drug (due to the absence of TPRV4 functionality), dose response tests for 12.5–75 µM HC-067047 was conducted on explants in the absence of impact (Figure 5c). No significant differences in the levels of cell death were observed in any of the doses of HC-067047 when stained for viability at 72 h post-addition of the drug (Figure 5d), suggesting that the cell death observed in Figure 5a was not due to any pharmacological effect.

### 2.7. The Mechanosensitive Ion Channel Blocker GsMTx4 Reduces Impact-Induced Cell Death

**Figure 6:**
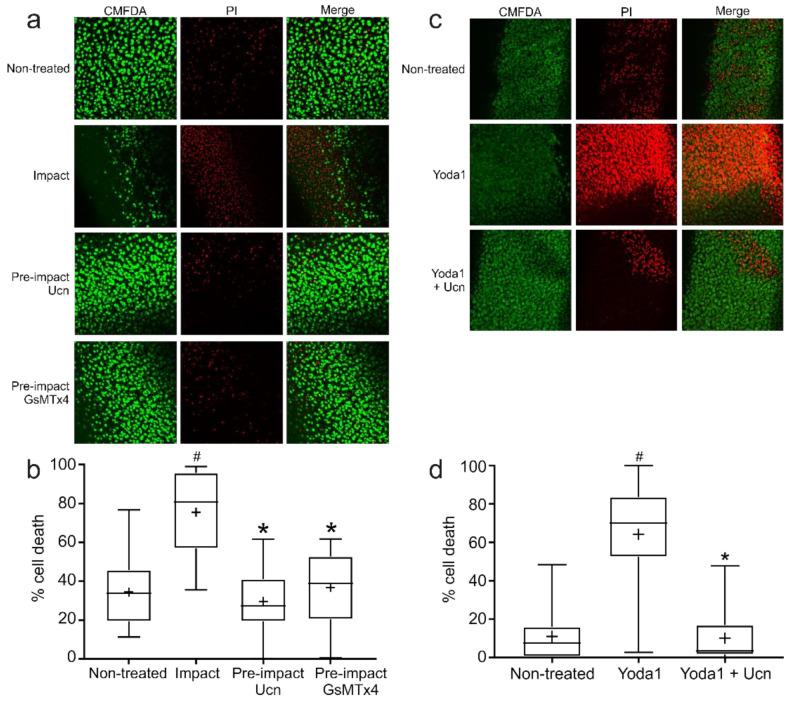
**Chondroprotection by Ucn involves the prevention of Ca^2+^ overload by Piezo1.** (**a**) Representative images of explants 72 h following impact with or without pre-treatment with Ucn or GsMTx4. Live cells stained with CMFDA (green) and dead cells with PI (red). (**b**) Quantification of percentage cell death from impact experiments shown in (**a**) (*n* = 6 explants, 5 fields of view/explant; # = *p* < 0.01 vs. non-treated, * = *p* < 0.01 vs. impact). (**c**) Representative images of explants 72 h after treatment with Yoda1 +/− Ucn. Live cells stained with CMFDA (green) and dead cells with PI (red). Representative images of *n* = 6 experiments, 5 fields of view/explant. (**d**) Quantification of percentage cell death from experiments shown in (**c**) (*n* = 6 explants, 5 fields of view/explant; # = *p* < 0.01 vs. non-treated, * = *p* < 0.01 vs. Yoda alone).

The previous calcium influx experiments demonstrated a role of Ucn in the modulation of a non-selective cation channel post-impact in order to reduce the excessive calcium influx observed in impacted explants alone. Given that the stimulus to chondrocyte damage in this case was mechanical trauma, the protective effect of the mechanosensitive ion channel blocker GsMTx4 was used alongside Ucn to investigate whether they had the same protective capabilities (Figure 6a). Explants pre-treated with either Ucn or GsMTx4 were able to significantly decrease chondrocyte cell death when compared to impact explants alone (Figure 6b), suggesting a link between Ucn protection and over-activation of a mechanosensitive ion channel following impact.

### 2.8. The Piezo1-Specific Activator Yoda1 Increases Chondrocyte Cell Death in the Absence of Impact

To study the role of Piezo1 in more detail in this system, explants were treated with the Piezo1-specific activator Yoda1 in the absence of impact (Figure 6c). Yoda1-treated explants exhibited high levels of cell death 72 h post-addition. Importantly, this Yoda1-induced cell death was significantly reduced by co-culture of these explants with Ucn (Figure 6d). Taken together, these results suggest that Ucn is affecting the same target as Yoda1 and therefore, Ucn is able to modulate Piezo1 in response to impact in order to reduce chondrocyte cell death.

## 3. Discussion

Injurious impact to cartilage results in the initiation of cell death pathways in chondrocytes and the accumulation of dead chondrocytes and their remnants promotes matrix catabolism [10,11]. A deficiency of healthy chondrocytes in the tissue also results in a lack of maintenance of the cartilage. The production of ECM components is reduced and normal turnover of the tissue is dysregulated, leading to a shift in the balance towards excess degradation over synthesis. Any further impact on the cartilage will also cause more damage, as chondrocytes are no longer capable of responding to this in a reparative capacity. Over time, this leads to an irreversible breakdown of cartilage and the onset of PTOA [30]. Current treatments of PTOA target the symptoms of the disease rather than the cause, and a lack of preventative approaches or successful non-invasive interventions will often result in the need for costly and invasive joint replacement surgery.

As such, there is a need for a chondroprotective agent that can be administered within a short time frame after injury in order to promote chondrocyte health and prevent progression to a catabolic, diseased tissue environment. We have previously demonstrated that the small, 40 amino acid peptide Ucn is an essential chondrocyte pro-survival factor [13] and in the current study have further shown it has a chondroprotective role in cartilage explants in response to mechanical overload. The chondroprotective pathway involves the modulation of a mechanosensitive cation channel, Piezo1, thus restricting excessive extracellular calcium influx to the cells. The relationship was shown to be specific to Piezo1 over other ion channels, specifically mechanosensitive TRPV4.

A simple drop tower model of impact was employed, similar to that widely used in previous studies [3,21,22,31]. The model does not allow quantification of the exact force or pressure exerted by the impact as it is not possible to calculate the displacement of the explant upon impact due to a lack of sensor underneath it. However, the impact energy transferred to the explants can be calculated using the formula *Ek* = *mgh*, where *Ek* = kinetic energy, *m* = mass of falling object, *g* = Earth’s gravitational acceleration and *h* = height of impact. Using a drop height of 5 cm and a 500 g weight resulted in an impact energy of 0.245 J. This is similar to that used elsewhere in the literature to induce OA-like changes in articular cartilage explants [23,31,32] and, while lower than that seen in the human joint during injury, the explants are not protected by the joint capsule, meaning much lower energies are needed to elicit an effect [33,34].

Porcine samples from young, healthy pigs were utilised rather than human tissue due to logistical difficulties in obtaining healthy human cartilage. Porcine cartilage is widely used in the field of OA research as it shares many characteristics with human cartilage in terms of structure, thickness and the anatomy of the joint [35], and the tissue also undergoes similar age-related osteoarthritic changes as human tissue [36]. However, the curved nature of the cartilage resulted in difficulties while imaging explants under confocal microscopy. It is also likely that the loading force was not homogeneously distributed across the whole explant. To overcome this, both macro and confocal microscopy techniques were used to image the whole explant. A five-image, defined mark-and-find approach was used for each set of experiments with the higher-power confocal microscope. This provided assurance that operator bias was eliminated, and that the average of all five images would prevent any skewing of the results.

The calcium influx observed was reduced by pre- and even post-impact addition of Ucn, and with the use of non-selective cation channel blocker Gd^3+^. Further exploration with mechanosensitive ion channel blockers highlighted Piezo1 as the potential channel at work in this system, as Piezo1-specific agonist Yoda1 was able to produce similar levels of cell death which could further be rescued by Ucn. Ucn is able to bind both CRF-R1 and R2, with a slight preference for CRF-R2 [24]. The CRF-R1 agonist CRF was able to provide chondroprotection whereas the CRF-R2 agonist Ucn2 was not, suggesting that Ucn interacts with CRF-R1 to induce this chondroprotection. The protection seen with CRF and Ucn was not significantly different, despite CRF having a greater affinity for CRF-R1. This suggests that the interaction between Ucn and CRF-R1 in this case is specific to this protection.

Antagonists of CRF-R1 (CP) and CRF-R2 (Ast2B) were also used pre-impact to identify the receptor at work in this system. Both CP and Ast2B-treated explants exhibited a significant increase in levels of cell death compared to non-treated explants. However, Ast2B-treated explants showed significantly fewer dead cells that CP-treated explants, suggesting that blocking CRF-R2 did not elicit as much damage as CRF-R1. The significant levels of cell death in Ast-2B-treated explants could suggest a role of Ucn binding to CRF-R2 in some cases to elicit chondroprotection. It is possible, therefore, that some compensation occurs between CRF-R1 and CRF-R2 when the explant is experiencing extreme stress in the form of impact. However, the results shown with Ucn2 suggest that Ucn binding CRF-R2 is not sufficient to significantly reduce levels of cell death.

Having established the interaction between Ucn and its receptor, the influence of calcium was investigated using real-time microscopy immediately after impact. Both pre- and post-impact, Ucn was able to significantly reduce calcium influx at a 1 h time point, as was treatment with Gd^3+^. Chondrocytes express a range of channels to regulate calcium in stress and non-stressful situations, including ligand-gated [37,38], voltage-dependent [39,40] and other non-selective channels [1,41,42]. The Gd^3+^ results indicate a non-selective ion channel, with mechanosensitive ion channels being the most likely candidates. We thus utilised GsMTx4, a potent blocker of cationic mechanosensitive ion channels [28], along with the more specific ion channel modifiers HC-067047 (for TRPV4) and Yoda1 (for Piezo1) to confirm the ion channel at work in this system.

TRPV4 and Piezo1 are well-discussed in chondrocyte literature [17] and we have previously found an essential role for Piezo1 in Ucn chondroprotection in the context of pharmacologically induced cell death [13]. Ucn has also been shown to exhibit chondroprotective properties in a trauma model of PTOA [1,43]. TRPV4 is an osmotically and mechanosensitive ion channel that has been shown to be an essential chondroprotective factor in age-related OA [43]. However, TPRV4 knockout has no effect on the cartilage of mice that underwent a destabilised medial meniscus model of PTOA, suggesting a distinct role of TRPV4 between the two phenotypes [44]. Our data demonstrate no role for TRPV4 in chondroprotection following single impact, as the TRPV4-specific blocker HC-067047 was unable to reduce cell death following trauma. These findings, presented within the context of current literature, confirm the distinct roles of these two mechanosensitive channels in the context of disease, with Piezo1 being more important in the context of PTOA prevention.

Our findings, particularly that Ucn can elicit its chondroprotective effects even after impact has occurred, highlight a potential clinical use for Ucn in the prevention of PTOA through delivery shortly after joint injury. However, as Ucn is expressed in many other tissues, systemic administration may elicit effects in other tissues. This is particularly pertinent with regard to the effects of Ucn on the cardiovascular system, where it is known to reduce blood pressure due to its properties as a vasodilator [45,46]. To avoid these effects, a controlled release Ucn delivery system locally administered directly to the joint could be ideal. Alternatively, identification of other components of this pro-survival pathway downstream that are more specific to the cells and tissues of the joint could allow the development of a more targeted therapy. Identification of the cell death pathway initiated may provide this, as may searching in greater detail for a link between the binding of Ucn and its receptor and the resulting modulation of Piezo1. As yet, the exact mechanism by which Ucn can regulate Piezo1 activity is not known. It may be that there are cytoskeletal changes or protein activation downstream of the G-protein signalling cascade that are involved which are more specific for this interaction. By exploring this link in greater detail, a potential specific drug target may be found. Another potential alternative may be to search for a mimetic of Ucn which has the same pro-survival capabilities but is limited in its off-target effects. The small size of Ucn at 40 amino acids in length lends it very well to this, and a small molecule screen could be carried out to discover a new pharmacological agent [47].

Taken together, this study demonstrates that Ucn is a chondroprotective agent in the context of injurious mechanical overload. Its ability to provide chondrocyte cytoprotection post-trauma highlights its potential as a novel DMOAD to maintain cartilage health and prevent progression from injury to PTOA.

## 4. Materials and Methods

### 4.1. Porcine Cartilage Harvest and Culture

Female 8-week-old suckling pigs were sacrificed by an abattoir according to local guidelines no more than 3 h before cartilage collection. Lateral and medial patellofemoral condyles were collected from both knees and washed in PBS, and 6 mm diameter biopsy punches were taken using a Uni-Core system punch (GE Healthcare, Amersham, UK). Punch biopsies that showed any superficial damage were discarded. Thickness of the explants varied due to the anatomical location, with explants demonstrating a thickness no greater than 6 mm retained. Explants were cultured in 24-well plates (one explant/well) containing Dulbecco’s Modified Eagle Medium (Gibco, New York, NY, USA) with 10% (*v*/*v*) foetal calf serum (FCS) (Gibco, New York, NY, USA), supplemented with 1000 units/mL penicillin and 1 mg/mL streptomycin for the first 24 h, dropping to 100 units/mL and 100 µg/mL, respectively, after this time. Explants were maintained at 37 °C and 5% CO_2_, with media changes every 48 h.

### 4.2. Development of a Single-Impact Drop Tower Device

Cartilage explants were subjected to impact from a custom-made drop tower device capable of applying a 500 g weight under sterile conditions (adapted from [23]). Prior to impact, a 10 cm plate was filled with PBS, and the explant was set upon it and aligned directly underneath the tube. The weight was hoisted to the desired height of impact before being released onto the superficial zone of the explant. Explants were then carefully removed using rounded forceps and replaced back into their respective wells.

### 4.3. Explant Impact in the Presence of Peptides and Modifying Agents

Pre-impact treated explants were incubated at 37 °C and 5% CO_2_ in media containing the relevant recombinant peptides or modifying agents in 1% FCS media for 30 min prior to impact. Post-impact treated explants were placed into fresh media with the relevant recombinant peptides or modifying agents added 30 min following impact. All explants were incubated for a further 72 h with no further media changes prior to assay.

In order to test the role of Ucn in the prevention of chondrocyte cell death, explants were incubated with 0.1 µM human Ucn (Ucn1) (Tocris, Bristol, UK) or Ucn2 (GenScript, Piscataway, NJ, USA). To investigate the role of Ucn receptors, CRF (Tocris, Bristol, UK), CP-154526 (CP) (Tocris, Bristol, UK) and Astressin 2B (Ast2B) (Tocris, Bristol, UK) were used at 0.1 µM. For ion channel identification, Yoda1 (Tocris, Bristol, UK) and GsMTx4 (Alomone Labs, Jerusalem, Israel) were also used at 0.1 μM concentration. HC-067047 (Tocris, Bristol, UK) was used either at 50 µM concentration, or in a concentration from 12.5 µM to 75 µM. Gadolinium (Gd^3+^) (Tocris, Bristol, UK) was used for calcium experiments at 500 µM concentration.

### 4.4. Live:Dead Cell Staining

Media from all explants at 72 h post-impact were replaced with 1 mL of serum-free media containing 1 µM CellTracker Green CMFDA (live cell stain, Thermo Fisher, Dartford, UK) and incubated at 37 °C for 30 min. PI stain (dead cell stain, Sigma, Haverhill, UK) 0.5 µM was added and incubated for 1 min before visualisation to allow for penetration of the dye without compromising the number of live cells present in the explant.

### 4.5. Confocal Microscopy

Macro-confocal images were obtained with the Leica Macro-confocal TCS LSI at 1× magnification. Images were captured in a format of 1024 × 1024 pixels with a scanning speed of 600 Hz and z-stack steps of 10 µm. CMFDA was visualised using a 488 nm laser line; PI was visualised under sequential scanning using the 532 nm laser line.

All other confocal images were captured using an upright Leica SP8 multi-photon microscope. Explants were visualised using a 10× objective, with the superficial zone oriented towards the objective in a PBS-lined 30 mm plate. A 1024 × 1024 pixel image size was collected for each explant under a scanning speed of 700 Hz with a step size of 2.5 µm. CMFDA and PI were visualised simultaneously with a laser emission bandwidth of 484–555 nm. The gain of the 488 ATOF laser was adjusted to 10.0 as compensation between the two dyes. To avoid operator bias and to ensure the whole explant was represented, a mark-and-find protocol was used to define 5 fields of view for analysis over the explant, which was kept consistent for each explant during one experiment. Total z-stack range varied between images due to the natural curvature of the explant.

### 4.6. Image Analysis

Macro-confocal images were analysed using ImageJ software. Images were converted to 8-bit and area and integrated density measurements were collected for each CMFDA and PI image, excluding the edge to eliminate any possibility of edge effect. The ratio of CMFDA:PI integrated density was presented as a green:red reading.

All other confocal images were analysed using the Spots function of Imaris v9.64. Cell diameter was determined by taking an average of 10 cells from a single snapshot and kept consistent throughout the analysis of each experiment. Thresholds of fluorescence intensity were adjusted based on one representative image per experiment and kept consistent throughout the analyses. The number of spots observed in the green and red channels were interpreted as the total number of cells seen in the image, with one spot equivalent to one cell.

### 4.7. Lactate Dehydrogenase Assay

Lactate dehydrogenase (LDH) assays were conducted using the Pierce™ LDH Cytotoxicity Assay Kit (Thermo Fisher Scientific, Dartford, UK) according to the manufacturer’s instructions. At the 72 h time point, 50 µL media from each well was plated in duplicate into wells of a 96-well plate along with a sample lysed with the kit lysis buffer as a positive control of LDH release. Explants were weighed and final LDH readings were adjusted based on weight to compensate for differences in the height of the explant.

### 4.8. Calcium Staining

Explants were impacted as described and returned to normal culture conditions in 1% FCS media for 10 min. All explants were then placed into buffer (137 mM NaCl, 6 mM KCl, 10 mM HEPES buffer, 1 mM MgCl_2_, 7 mM glucose, 2.6 mM CaCl_2_, in dH_2_O at pH 7.4) containing 1 μM Fluo-4 AM and 5 μM Hoechst-333285 and placed under normal culture conditions in the dark. Post-impact Ucn- and Gd^3+^-treated explants were administered 0.1 μM Ucn or 500 μM Gd^3+^ 30 min post-impact. At 1 h post-impact, all explants were visualised using a Leica SP8 confocal microscope.

### 4.9. Data Analysis

Data from each experiment were collected into Microsoft Excel spreadsheets and collated on GraphPad Prism8. Data were tested for normality using a D’Agostino and Pearson normality test, then analysed using a one-way ANOVA with Tukey’s multiple comparisons test if normally distributed or Kruskal–Wallis with Dunn’s multiple comparisons test if not. Graphs in Figure 1a present mean values, with error bars showing SEM. Box and whisker plots show median and 25th and 75th percentiles. Mean is displayed with a ‘+’ on each plot, and vertical bars show minimum and maximum values.

## Data Availability

Data sharing not applicable as no datasets have been generated or analysed in this study.

## References

[1] Lee J.H., Fitzgerald J.B., Dimicco M.A., Grodzinsky A.J. (2005). Mechanical injury of cartilage explants causes specific time-dependent changes in chondrocyte gene expression. Arthritis Rheum..

[2] Natoli R.M., Scott C.C., Athanasiou K.A. (2008). Temporal effects of impact on articular cartilage cell death, gene expression, matrix biochemistry, and biomechanics. Ann. Biomed. Eng..

[3] Otsuki S., Brinson D.C., Creighton L., Kinoshita M., Sah R.L., D’Lima D., Lotz M. (2008). The effect of glycosaminoglycan loss on chondrocyte viability: A study on porcine cartilage explants. Arthritis Rheum..

[4] Tew S.R., Kwan A.P., Hann A., Thomson B.M., Archer C.W. (2000). The reactions of articular cartilage to experimental wounding: Role of apoptosis. Arthritis Rheum..

[5] Martel-Pelletier J., Barr A.J., Cicuttini F.M., Conaghan P.G., Cooper C., Goldring M.B., Goldring S.R., Jones G., Teichtahl A.J., Pelletier J.P. (2016). Osteoarthritis. Nat. Rev. Dis. Primers.

[6] Brown T.D., Johnston R.C., Saltzman C.L., Marsh J.L., Buckwalter J.A. (2006). Posttraumatic osteoarthritis: A first estimate of incidence, prevalence, and burden of disease. J. Orthop. Trauma.

[7] Gelber A.C., Hochberg M.C., Mead L.A., Wang N.Y., Wigley F.M., Klag M.J. (2000). Joint injury in young adults and risk for subsequent knee and hip osteoarthritis. Ann. Intern. Med..

[8] Khella C.M., Asgarian R., Horvath J.M., Rolauffs B., Hart M.L. (2021). An Evidence-Based Systematic Review of Human Knee Post-Traumatic Osteoarthritis (PTOA): Timeline of Clinical Presentation and Disease Markers, Comparison of Knee Joint PTOA Models and Early Disease Implications. Int. J. Mol. Sci..

[9] Musumeci G., Aiello F.C., Szychlinska M.A., Di Rosa M., Castrogiovanni P., Mobasheri A. (2015). Osteoarthritis in the XXIst century: Risk factors and behaviours that influence disease onset and progression. Int. J. Mol. Sci..

[10] Hashimoto S., Ochs R.L., Rosen F., Quach J., McCabe G., Solan J., Seegmiller J.E., Terkeltaub R., Lotz M. (1998). Chondrocyte-derived apoptotic bodies and calcification of articular cartilage. Proc. Natl. Acad. Sci. USA.

[11] Hashimoto S., Takahashi K., Amiel D., Coutts R.D., Lotz M. (1998). Chondrocyte apoptosis and nitric oxide production during experimentally induced osteoarthritis. Arthritis Rheum..

[12] Intekhab-Alam N.Y., White O.B., Getting S.J., Petsa A., Knight R.A., Chowdrey H.S., Townsend P.A., Lawrence K.M., Locke I.C. (2013). Urocortin protects chondrocytes from NO-induced apoptosis: A future therapy for osteoarthritis?. Cell Death Dis..

[13] Lawrence K.M., Jones R.C., Jackson T.R., Baylie R.L., Abbott B., Bruhn-Olszewska B., Board T.N., Locke I.C., Richardson S.M., Townsend P.A. (2017). Chondroprotection by urocortin involves blockade of the mechanosensitive ion channel Piezo1. Sci. Rep..

[14] Nourse J.L., Pathak M.M. (2017). How cells channel their stress: Interplay between Piezo1 and the cytoskeleton. Semin. Cell Dev. Biol..

[15] Wang Y., Xiao B. (2018). The mechanosensitive Piezo1 channel: Structural features and molecular bases underlying its ion permeation and mechanotransduction. J. Physiol..

[16] Du G., Li L., Zhang X., Liu J., Hao J., Zhu J., Wu H., Chen W., Zhang Q. (2020). Roles of TRPV4 and piezo channels in stretch-evoked Ca^2+^ response in chondrocytes. Exp. Biol. Med..

[17] Servin-Vences M.R., Richardson J., Lewin G.R., Poole K. (2018). Mechanoelectrical transduction in chondrocytes. Clin. Exp. Pharmacol. Physiol..

[18] Chu C.R., Szczodry M., Bruno S. (2010). Animal models for cartilage regeneration and repair. Tissue Eng. Part B Rev..

[19] Johnson C.I., Argyle D.J., Clements D.N. (2016). In vitro models for the study of osteoarthritis. Vet. J..

[20] Smeriglio P., Lai J.H., Yang F., Bhutani N. (2015). 3D Hydrogel Scaffolds for Articular Chondrocyte Culture and Cartilage Generation. J. Vis. Exp..

[21] Delco M.L., Bonnevie E.D., Szeto H.S., Bonassar L.J., Fortier L.A. (2018). Mitoprotective therapy preserves chondrocyte viability and prevents cartilage degeneration in an ex vivo model of posttraumatic osteoarthritis. J. Orthop. Res..

[22] Ding L., Guo D., Homandberg G.A., Buckwalter J.A., Martin J.A. (2014). A single blunt impact on cartilage promotes fibronectin fragmentation and upregulates cartilage degrading stromelysin-1/matrix metalloproteinase-3 in a bovine ex vivo model. J. Orthop. Res..

[23] Huser C.A., Davies M.E. (2007). Calcium signaling leads to mitochondrial depolarization in impact-induced chondrocyte death in equine articular cartilage explants. Arthritis Rheum..

[24] Vaughan J., Donaldson C., Bittencourt J., Perrin M.H., Lewis K., Sutton S., Chan R., Turnbull A.V., Lovejoy D., Rivier C. (1995). Urocortin, a mammalian neuropeptide related to fish urotensin I and to corticotropin-releasing factor. Nature.

[25] Facci L., Stevens D.A., Pangallo M., Franceschini D., Skaper S.D., Strijbos P.J. (2003). Corticotropin-releasing factor (CRF) and related peptides confer neuroprotection via type 1 CRF receptors. Neuropharmacology.

[26] Gavenis K., Schumacher C., Schneider U., Eisfeld J., Mollenhauer J., Schmidt-Rohlfing B. (2009). Expression of ion channels of the TRP family in articular chondrocytes from osteoarthritic patients: Changes between native and in vitro propagated chondrocytes. Mol. Cell Biochem..

[27] Jin L., Li C., Li R., Sun Z., Fang X., Li S. (2014). Corticotropin-releasing hormone receptors mediate apoptosis via cytosolic calcium-dependent phospholipase A(2) and migration in prostate cancer cell RM-1. J. Mol. Endocrinol..

[28] Suchyna T.M. (2017). Piezo channels and GsMTx4: Two milestones in our understanding of excitatory mechanosensitive channels and their role in pathology. Prog. Biophys. Mol. Biol..

[29] Suchyna T.M., Johnson J.H., Hamer K., Leykam J.F., Gage D.A., Clemo H.F., Baumgarten C.M., Sachs F. (2000). Identification of a peptide toxin from Grammostola spatulata spider venom that blocks cation-selective stretch-activated channels. J. Gen. Physiol..

[30] Little C.B., Hunter D.J. (2013). Post-traumatic osteoarthritis: From mouse models to clinical trials. Nat. Rev. Rheumatol..

[31] Huser C.A., Davies M.E. (2006). Validation of an in vitro single-impact load model of the initiation of osteoarthritis-like changes in articular cartilage. J. Orthop. Res..

[32] Huser C.A., Peacock M., Davies M.E. (2006). Inhibition of caspase-9 reduces chondrocyte apoptosis and proteoglycan loss following mechanical trauma. Osteoarthr. Cartil..

[33] Clements K.M., Bee Z.C., Crossingham G.V., Adams M.A., Sharif M. (2001). How severe must repetitive loading be to kill chondrocytes in articular cartilage?. Osteoarthr. Cartil..

[34] Verteramo A., Seedhom B.B. (2007). Effect of a single impact loading on the structure and mechanical properties of articular cartilage. J. Biomech..

[35] Taylor S.D., Tsiridis E., Ingham E., Jin Z., Fisher J., Williams S. (2012). Comparison of human and animal femoral head chondral properties and geometries. Proc. Inst. Mech. Eng. H.

[36] Macfadyen M.A., Daniel Z., Kelly S., Parr T., Brameld J.M., Murton A.J., Jones S.W. (2019). The commercial pig as a model of spontaneously-occurring osteoarthritis. BMC Musculoskelet. Disord..

[37] He Z., Leong D.J., Zhuo Z., Majeska R.J., Cardoso L., Spray D.C., Goldring M.B., Cobelli N.J., Sun H.B. (2016). Strain-induced mechanotransduction through primary cilia, extracellular ATP, purinergic calcium signaling, and ERK1/2 transactivates CITED2 and downregulates MMP-1 and MMP-13 gene expression in chondrocytes. Osteoarthr. Cartil..

[38] Mobasheri A., Matta C., Uzieliene I., Budd E., Martin-Vasallo P., Bernotiene E. (2019). The chondrocyte channelome: A narrative review. Jt. Bone Spine.

[39] Matta C., Zakany R., Mobasheri A. (2015). Voltage-dependent calcium channels in chondrocytes: Roles in health and disease. Curr. Rheumatol. Rep..

[40] Srinivasan P.P., Parajuli A., Price C., Wang L., Duncan R.L., Kirn-Safran C.B. (2015). Inhibition of T-Type Voltage Sensitive Calcium Channel Reduces Load-Induced OA in Mice and Suppresses the Catabolic Effect of Bone Mechanical Stress on Chondrocytes. PLoS ONE.

[41] O’Conor C.J., Leddy H.A., Benefield H.C., Liedtke W.B., Guilak F. (2014). TRPV4-mediated mechanotransduction regulates the metabolic response of chondrocytes to dynamic loading. Proc. Natl. Acad. Sci. USA.

[42] Song T., Ma J., Guo L., Yang P., Zhou X., Ye T. (2017). Regulation of chondrocyte functions by transient receptor potential cation channel V6 in osteoarthritis. J. Cell Physiol..

[43] Clark A.L., Votta B.J., Kumar S., Liedtke W., Guilak F. (2010). Chondroprotective role of the osmotically sensitive ion channel transient receptor potential vanilloid 4: Age- and sex-dependent progression of osteoarthritis in Trpv4-deficient mice. Arthritis Rheum..

[44] O’Conor C.J., Ramalingam S., Zelenski N.A., Benefield H.C., Rigo I., Little D., Wu C.L., Chen D., Liedtke W., McNulty A.L. (2016). Cartilage-Specific Knockout of the Mechanosensory Ion Channel TRPV4 Decreases Age-Related Osteoarthritis. Sci. Rep..

[45] Davis M.E., Pemberton C.J., Yandle T.G., Lainchbury J.G., Rademaker M.T., Nicholls M.G., Frampton C.M., Richards A.M. (2005). Effect of urocortin 1 infusion in humans with stable congestive cardiac failure. Clin. Sci..

[46] Parkes D.G., Vaughan J., Rivier J., Vale W., May C.N. (1997). Cardiac inotropic actions of urocortin in conscious sheep. Am. J. Physiol..

[47] Janzen W.P. (2014). Screening technologies for small molecule discovery: The state of the art. Chem. Biol..

